# Prognostic Significance of Elevated Cholestatic Enzymes for Fibrosis and Hepatocellular Carcinoma in Hospital Discharged Chronic Viral Hepatitis Patients

**DOI:** 10.1038/s41598-017-11111-5

**Published:** 2017-08-31

**Authors:** Wen Xie, Yang Cao, Min Xu, Jiangbin Wang, Changyu Zhou, Xingxiang Yang, Xiaoxia Geng, Wenhong Zhang, Ning Li, Jun Cheng

**Affiliations:** 1 Department of Infectious Diseases, Beijing Ditan Hospital, Capital Medical University, Beijing, China; 2 Department of Infectious Diseases, Guangzhou Eighth People’s Hospital, Guangzhou, China; 3 Department of Infectious Diseases, China-Japan Union Hospital, Changchun, China; 4 Department of Infectious Diseases, Sichuan Provincial People’s Hospital, Chengdu, China; 5 Department of Infectious Diseases, Huashan Hospital, Fudan University, Shanghai, China

## Abstract

Chronic viral hepatitis (CVH) is the root cause of liver fibrosis and subsequent hepatocellular carcinoma (HCC). We conducted a cross-sectional, observational study based on medical records and primary data collection from patients with CVH who were admitted in five hospitals across China between February and September 2013 to determine the prevalence of elevated cholestatic enzymes (ALP and/or GGT above ULN) in discharged Chinese patients with CVH as a primary outcome and secondarily evaluated the relationship of these enzymes with fibrosis and disease severity. Majority of the patients (56%) had cholestatic enzyme elevation at discharge, with high prevalence of liver fibrosis (37.6% vs. 20.1%, p < 0.001) and cirrhosis (Child-Pugh B: 56.9% vs. 48.7%; Child-Pugh C: 17.4% vs. 12.5%; p < 0.001) in addition to significantly higher odds of liver fibrosis (OR 1.54; p = 0.024) and a trend towards higher odds of moderate-to-severe cirrhosis (OR 1.24; p = 0.317) compared to those who had normal enzyme levels. Elevated cholestatic enzyme levels serve as important prognosticators of liver fibrosis in CVH patients. Therefore, pre-discharge testing of cholestatic enzymes is recommended to identify CVH patients and provide prophylactic care.

## Introduction

Viral hepatitis poses a major public health problem throughout the world. Most prevalent hepatitis B and C infections lead to chronic disease in over 500 million people worldwide^1, 2^, accounting for 1.4 million deaths in 2010^3^. It is estimated that HBV and HCV is the root cause of about 80% of all hepatocellular carcinomas (HCC) by promoting cirrhosis^4, 5^ which significantly reduced the life expectancy of the infected patients^6, 7^. Most patients are asymptomatic in the early stage as specific clinical symptoms often occur at advanced disease stages, which are usually irreversible^8^. Hence, the prognosis of the infection to liver disease is very crucial. Abnormal liver enzyme levels (Alanine aminotransferase, ALT) may signal liver damage due to cirrhosis, fibrosis or alteration in bile flow^9^. However, recent studies show that ALT is not a reliable marker of liver disease as the serum ALT levels are not too high in chronic hepatitis as in acute hepatitis and that the ALT level increase in hospitalised patients due to the hospital food and restricted physical activity^9^. Hence, researchers suggest the physicians that ALT is a standard sign of liver inflammation, but not a good reflection of fibrosis progression^10^. At this point monitoring the biomarkers specific for fibrosis is required. Recent studies demonstrate that biomarkers of cholestasis (which lead to hepatocellular injury, bile duct obstruction and ultimately fibrosis) including increased levels of serum alkaline phosphatase (ALP) and gamma glutamyltranspeptidase (GGT), as well as conjugated hyperbilirubinemia which occurred in more advanced stages are related to pathological changes and poor prognosis in liver diseases^11–13^. Furthermore, intervention studies reported amelioration of abnormal cholestasis biomarkers and alleviation of clinical symptoms in patients who received treatment for intrahepatic cholestasis^14, 15^. These enzymes are also known to be increased in chronic viral hepatitis^16^. The presence of these enzymes at the time of discharge from hospital may signal a risk of fibrosis, cirrhosis and ultimately HCC. Though there are gold standard methods such as liver biopsy^9^ and biomarkers such as serum hyaluronic acid (HA) and type IV collagen for the detection of liver fibrosis^10–13^, in addition to the modified Child-Pugh classification system commonly used to determine disease severity and overall prognosis in patients with chronic liver disease^14^, monitoring the serum level of cholestatic enzymes may warn the risk of future fibrosis at the time of discharge and makes the follow-up and post-discharge management feasible. In China, the decision to discharge a patient with CVH from the hospital is clinically judged based on improvement in visible clinical signs and symptoms rather than biomarker monitoring and therefore, patients with increased ALP and/or GGT levels may not be identified and are discharged without appropriate follow-up and treatment. Despite the association of the cholestatic enzymes with the prognosis of liver diseases, there is limited evidence from the hospital setting at the time of discharge. Understanding the relationship of cholestatic enzymes with disease severity and prognosis in inpatients with CVH may guide disease management during and after discharge. The present study elucidates the association between the elevated cholestatic enzymes levels at discharge with the prognosis of liver fibrosis and disease severity in Chinese CVH patients.

## Results

### Patient characteristics and prevalence of cholestatic enzyme elevation

A total of 1000 patients were enrolled in the study. Two patients did not complete the study visits for examination of laboratory indices and hence, were excluded from the final analysis. The remaining 998 patients (male, n = 723 (72.4%); mean age: 47 ± 14 years) were included in the analysis of whom 84.4% (n = 842) had hepatitis B, 14.7% (n = 147) had hepatitis C, and 0.9% (n = 9) had dual infection. Table 1 shows the demographic and clinical characteristics of the analyzed population. The median duration of CVH at the time of admission was 6 (0‒56) years and about two-fifth of the patients had CVH for at least 10 years. Cholestatic enzyme elevation (ALP and/or GGT) was present in 56.2% of patients at discharge (Table 1). Abnormalities in other liver function tests (LFTs) such as alteration of bile acid flow and total bilirubin were observed in 58.7% and 55.5% patients respectively. Indicators of hepatocellular damage, AST and ALT were observed in 49.5% and 44.1% patients, whereas, indicators of liver function, high albumin level and prothrombin time were observed in 37.9% and 35.6% patients respectively. Fibrosis was present in 29.4% of the patients. About one-third (30.6%) of patients had Child-Pugh A cirrhosis, while 53.8% had Child-Pugh B cirrhosis and 15.6% had Child-Pugh C cirrhosis (Table 1).

**Table 1:** Patient demographics and characteristics.

Variable	Overall (n = 998)
Age (years), mean ± SD	47 ± 14
Male	723 (72.4)
Duration of CVH at admission (years)	6 (0‒56)
**<**5 years	458 (45.9)
5 to 9 years	155 (15.5)
≥10 years	385 (38.6)
ALP (U/L)	101.0 (16.0–3243.0)
GGT (U/L)	59.0 (7.0–966.0)
Abnormal ALP and/or GGT	561 (56.2)
Abnormal ALP	255 (25.6)
Abnormal GGT	463 (46.4)
Abnormal ALP and GGT	158 (15.9)
Bile acid (μmol/L)	16.8 (0.2–593.0)
Total bilirubin (μmol/L)	22.4 (4.1–544.9)
AST (U/L)	39.3 (7.0–936.0)
ALT (U/L)	36.7 (5.0–2585.0)
Albumin (g/L)	36.0 (16.0–64.0)
Prothrombin time (secs)	13.3 (7.5–168.0)
HA (ng/ml)	100.8 (0.8‒2960.0)
Type IV collagen (ng/ml)	98.2 (3.5‒2265.0)
**Child-Pugh score**	
A	244 (30.6)
B	429 (53.8)
C	124 (15.6)

Results are presented as median (min–max) or n (%) unless otherwise specified. ALP, alkaline phosphatase; ALT, alanine transaminase; AST, aspartate transaminase; CVH, chronic viral hepatitis; GGT, gamma glutamyltransferase; HA, hyaluronic acid.

### Patient characteristics of patients divided based on cholestatic enzyme status

Patient demographics, characteristics, and liver function profile summarized according to cholestatic enzyme status are presented in Table 2. Patients who had cholestatic enzyme elevation were younger than those who had normal levels of cholestatic enzymes (47 ± 14 vs. 49 ± 14 years; p = 0.025). The elevation of these enzymes was predominant in female patients compared to normal levels (36.4% vs. 20.7%; p < 0.001). Patients who had cholestatic enzyme elevation also exhibited significantly higher abnormality rates in other LFTs [bile acid (70.7% vs. 43.2%), total bilirubin (36.7% vs. 17.4%), AST (65.2% vs. 29.3%), ALT (57.9% vs. 26.3%), albumin (42.3% vs. 32.3%), and prothrombin time (40.3% vs. 29.7%)] compared with those who had normal levels (p < 0.001 for all). The distribution of CVH duration was similar in both groups (Table 2). Patients with abnormal ALP or GGT had higher abnormal HA and type IV collagen. The association between abnormal ALP or GGT with abnormal HA and type IV collagen was statistically significant compared to the normal ALP or GGT, P = 0.0001. Comparisons of OR between Cholestatic enzymes and prothrombin was not statistically significant (P = 0.06990).

**Table 2:** Univariate comparison of patient demographics, characteristics, and liver function profile by cholestatic enzyme status.

Variable	Elevated ALP and/or GGT	Normal ALP and GGT)	^*^p-value
Age (years)	47 ± 14	49 ± 14	**0.025**
Gender			**<0.001**
Male	278 (63.6)	444 (79.3)	
Female	159 (36.4)	116 (20.7)	
Duration of CVH			0.591
** <**5 years	262 (46.8)	196 (44.9)	
5 to 9 years	90 (16.1)	65 (14.0)	
≥10 years	208 (37.1)	176 (40.3)	
Bile acid			**<0.001**
Normal	164 (29.3)	248 (56.8)	
Abnormal	396 (70.7)	189 (43.2)	
Total bilirubin			**<0.001**
Normal and mild abnormal (≤2 ULN^†^)	351 (63.2)	356 (82.6)	
Moderate abnormal (>2 to 5 ULN^†^)	109 (19.6)	52 (12.1)	
Severe abnormal (>5 ULN^†^)	95 (17.1)	23 (5.3)	
AST			**<0.001**
Normal	195 (34.8)	309 (70.5)	
Abnormal	365 (65.2)	128 (29.3)	
ALT			**<0.001**
Normal	236 (42.1)	322 (73.7)	
Abnormal	324 (57.9)	115 (26.3)	
Albumin			**<0.001**
Normal	323 (57.7)	296 (67.7)	
Abnormal	237 (42.3)	141 (32.3)	
Prothrombin			**<0.001**
Normal	334 (59.7)	306 (70.3)	
Abnormal	225 (40.3)	129 (29.7)	

Results are presented as mean ± SD or n (%).

^*^Comparison between patients who had elevated ALP and/or GGT with those who had normal levels.

^†^ULN of the local laboratory.

ALP, alkaline phosphatase; ALT, alanine transaminase; AST, aspartate transaminase; CVH, chronic viral hepatitis; GGT, gamma glutamyltransferase; ULN, upper limit of normal.

### Relationship between cholestatic enzyme elevation and clinical outcomes

**Figure 1:**
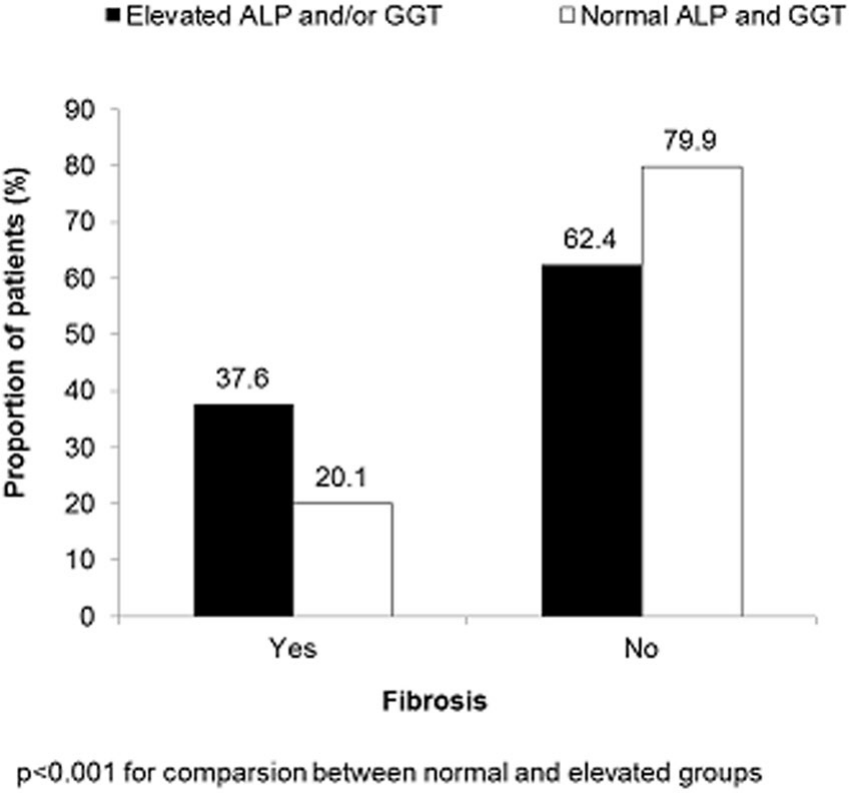
Distribution of patients with liver fibrosis based on cholestatic enzyme elevation.

**Figure 2:**
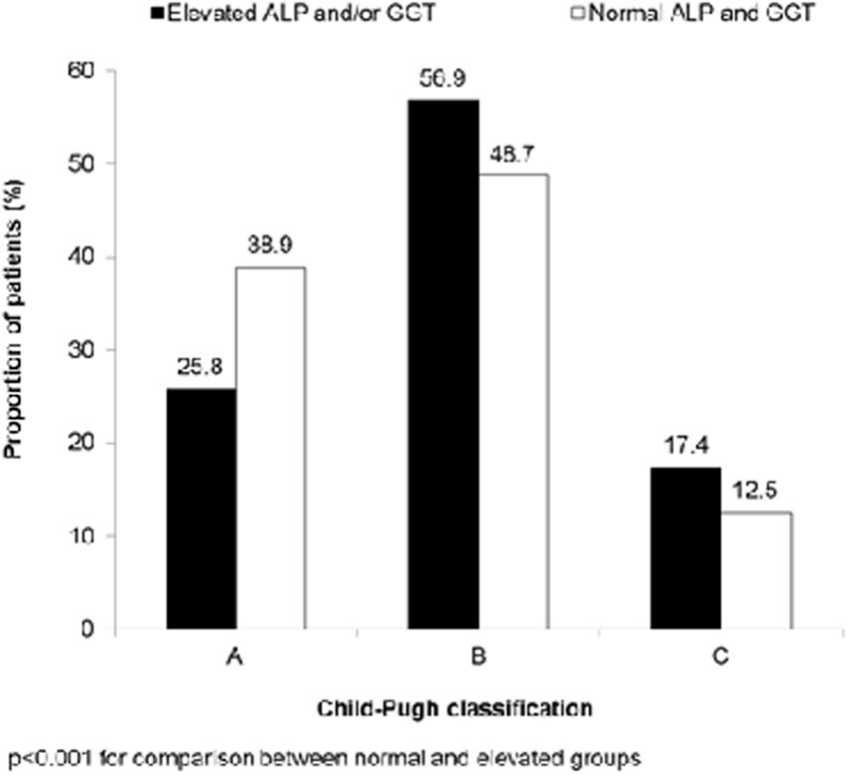
Distribution of patients with Child-Pugh (**A**,**B** and **C**) stage liver disease based on cholestatic enzyme elevation.

The proportion of patients with elevated and normal ALP and/or GGT contributing to fibrosis and liver disease severity is shown in Figs 1 and 2 respectively. Patients who had cholestatic enzyme elevation were associated with a significantly higher rate of liver fibrosis than those who exhibited normal levels of cholestatic enzymes (37.6% vs. 20.1%; p < 0.001) (Fig. 1). There were also more patients with compromised hepatic function (Child-Pugh B 56.9% vs. 48.7%) or decompensated disease (Child-Pugh C 17.4% vs. 12.5%) in patients who had cholestatic enzyme elevation compared with those who had normal levels of these enzymes (p < 0.001) (Fig. 2). In multivariate analyses adjusted for potential confounders, patients with cholestatic enzyme elevation were associated with significantly higher odds of liver fibrosis (odds ratio [OR] 1.54; p = 0.024) and non-significantly higher odds of advanced cirrhosis (OR 1.24; p = 0.317) compared with patients who had normal levels of cholestatic enzymes (Table 3). Abnormalities in bile acid (OR 2.09; p < 0.001), total bilirubin (moderate abnormal OR 2.23; p < 0.001, severe abnormal OR 3.90; p < 0.001), AST (OR 1.88; p = 0.003), albumin (OR 1.46; p = 0.04), and prothrombin time (OR 2.46; p < 0.001) were also significantly associated with higher odds of liver fibrosis. In addition, raised bile acid was associated with higher odds of advanced liver disease or HCC (Child-Pugh; OR 4.21; p < 0.001), whereas abnormal ALT was not associated with higher odds of liver fibrosis and advanced liver disease or HCC (OR 0.53; p = 0.004 and OR 0.49; p = 0.004, respectively) (Table 3). The was no correlation between ALT and child-Pugh score (P = 0.0024). Univariate logistic regression model revealed that abnormal HA and type IV collagen were associated with abnormal ALP or GGT (OR: 2.396 (CI: 1.771–3.244), P < 0.0001) and higher odds of Child-Pugh score (Child-Pugh B vs A, OR: 5.053 (3.229–7.907, P < 0.0001 and Child-Pugh score C vs A, OR:10.21 (5.904–17.654, P < 0.0001). On multivariate regression analysis in predicting the risk factors for abnormal ALP or GGT revealed that patients with abnormal Bile acid, ALT and AST demonstrated higher odds of abnormal ALP or GGT (OR:2.338 [1.653–3.3.7], P < 0.0001; OR:2.201 [1.522–3.184], P < 0.0001; OR:2.187 [1.518–3.150], P < 0.0001, respectively) also patients with abnormal HA and abnormal type IV collagen had higher odds of abnormal ALP or GGT (OR:1.441) with marginal P-value (0.0562), whereas PT, albumin, Course of CVH did not show association with abnormal ALP or GGT (OR:0.751 [0.515–1.094], P = 0.135; OR:1.115 [0.795–1.564], P = 0.52); OR:0.931 [0.599–1.447], P = 0.749, respectively).

**Table 3:** Adjusted association between cholestatic enzyme elevation and liver fibrosis or disease severity.

	Liver fibrosis		p-value	Child-Pugh B and C		p-value
	OR	95% CI		OR	95% CI	
Cholestatic enzyme elevation (yes vs. no)	1.54	1.06‒2.24	**0.024**	1.24	0.82‒1.88	0.317
Age (years)	1.00	0.99‒1.01	0.974	1.04	1.03‒1.06	**<0.001**
Gender (male vs. female)	1.22	0.82‒1.81	0.337	1.68	1.10‒2.58	**0.017**
Duration of CVH (years)	—	—	0.051	0.98	0.96‒1.00	0.060
5 to 9 years vs.**<**5 years	0.88	0.54‒1.42	0.594	—	—	
10 years vs.**<**5 years	0.63	0.43‒0.92	0.016	—	—	
Bile acid^*^	2.09	1.37‒3.18	**<0.001**	4.21	2.78‒6.36	**<0.001**
Total bilirubin			**<0.001**	—	—	
Moderate abnormal vs. mild abnormal and normal	2.23	1.45‒3.44	**<**0.001	—	—	
Severe abnormal vs. mild abnormal and normal	3.90	2.28‒6.67	**<**0.001	—	—	
AST^*^	1.88	1.23‒2.85	**0.003**	1.48	0.91‒2.41	0.118
ALT^*^	0.53	0.35‒0.81	**0.004**	0.49	0.31‒0.80	**0.004**
Albumin^*^	1.46	1.02‒2.10	**0.04**	—	—	
Prothrombin^*^	2.46	1.70‒3.56	**<0.001**	—	—	
Liver fibrosis (yes vs. no)	—	—	—	3.39	2.11‒5.45	**<0.001**

^*^Abnormal vs. normal.

ALT, alanine transaminase; AST, aspartate transaminase; CVH, chronic viral hepatitis.

## Discussion

Liver biopsy has been traditionally used to determine the risk of fibrosis and disease progression in chronic viral hepatitis. However, the procedure involves discomfort and risks to the patient affecting the quality of life^17^, warranting non-invasive methods. Recent studies have reported the role of cholestatic enzymes GGT and ALP in fibrosis and suggested that these enzymes could serve as potential biomarkers for fibrosis^18^. However, association of ALT, AST, and GGT values with the HAI in patients with CVH remains obscure^19^. Despite the elevation in cholestatic enzyme levels in CVH, they were never been used diagnostically for fibrosis in hospital discharged CVH patients in China. Rather, patients are discharged based on the improvement in clinical signs and symptoms and sufficient resolution of the medical cause for hospitalisation, with no standardised discharge criteria used across hospitals. Moreover, the decision to discharge is also influenced by non-medical reasons, such as availability of hospital beds, patient’s affordability. Hence, patients with CVH who have increased ALP and/or GGT levels may not be identified and are discharged without appropriate follow-up and treatment in the real-world hospital setting. In our cohort of Chinese inpatients who are diagnosed with chronic hepatitis B and/or C, the prevalence of elevated cholestatic enzymes (ALP and/or GGT) was high (56%) at the time of discharge. Also, patients who had cholestatic enzyme elevation were associated with a significantly higher rate of liver fibrosis than those who exhibited normal levels of cholestatic enzymes. Further, a high prevalence of Child-Pugh class B and C was observed in such patients. In addition, we found that cholestatic enzyme elevation was associated with increased odds of liver fibrosis and a trend towards increased odds of advanced cirrhosis. This is consistent with the findings of previous studies which indicate that abnormalities in GGT and/or ALP are related to the severity and prognosis of liver diseases. In a study conducted by Wang and colleagues, increase in GGT levels was found to be directly proportional to the liver histopathological changes in patients with chronic hepatitis^11^. In another study, elevated GGT level was associated with bile duct damage and fibrosis in patients with chronic liver disease^20^. Lopez *et al*. reported that abnormalities in GGT, ALP and other liver enzymes were related to more advanced disease stage in patients with hepatocellular carcinoma^13^. Jungst *et al*. reported that the occurrence of intrahepatic cholestasis in patients with chronic hepatitis B indicated severe progressive liver disease or an acute exacerbation of HBV infection. In patients with chronic hepatitis C infection, increased GGT levels were associated with more advanced liver diseases^21^. The goal of CVH management is to prevent or delay the progression of liver fibrosis or cirrhosis. In this study, the prevalence of elevated cholestatic enzymes was estimated based on elevations of ALP level, GGT level or both levels above the respective ULN rather than the European Association for the Study of the Liver (EASL)-proposed diagnostic criteria for cholestasis (ALP ≥ 1.5 ULN and GGT > 3 ULN)^22^. This EASL proposed criteria is more for primary cholestasis, but not the secondary cholestasis (eg. caused by viral hepatitis). So this study provided a unique opportunity to investigate whether early increase in ALP, GGT or both levels in patients with CVH warrant any early intervention. In a study with 70 chronic hepatitis B Chinese patients, GGT reflected inflammation in the liver better than ALT and AST proving that GGT alone can be an important role in the clinical evaluation of chronic hepatitis B^23^. In our study both GGT and ALP were found to be associated with prognosis of chronic hepatitis into liver disease. Our data show that active surveillance needs to be conducted even in asymptomatic patients who have increased ALP and/or GGT levels. In addition, this study also showed that raised bile acid was independently associated with both liver fibrosis and cirrhosis, Similar to a recent study which showed that serum bile acid levels were significantly higher in patients with severe liver fibrosis than those with non-severe liver fibrosis^24^. These findings propose bile acid levels in the prognostic assessment of patients with CVH before discharge. Given the high prevalence of elevated cholestatic enzymes and correlation with liver fibrosis and Child-Pugh disease severity observed in our cohort of discharged patients, our data suggest that ALP and/or GGT levels should be considered in the assessment and treatment of patients with CVH at the time of hospital discharge, along with clinical parameters and any other prognostic factors considered by their physicians.

Our findings however need to be interpreted in light of its limitations. First, the cross-sectional design of this study only provides information on the association between elevated cholestatic enzymes and liver fibrosis or disease severity and does not yield any conclusion on the pathological mechanisms behind the phenomena, warranting future longitudinal research studies. Secondly, the high prevalence rates of cholestatic enzyme levels in our cohort might be due to the under looked testing of serum levels at the time of discharge as per routine China clinical practice and lack of use of standard method like liver biopsy to provide insight to the correlation between the elevated ALP/GGT in detection of fibrosis is also limiting the accurate evidence Nevertheless, our results reflect the observation in real-world hospital settings in China, allowing recommendations from this study that are relevant for daily clinical practice to be formulated. Further, this study was conducted in major hospitals where patients had relatively more severe CVH. Therefore, the results may not be generalizable to the general population with mild CVH. Nonetheless, our study provided important evidence on the value of cholestatic enzymes in guiding disease management in a specific population of patients who are at relatively higher risk of disease progression.

To conclude, we suggest assessment of elevated cholestatic enzymes at the time of hospital discharge, along with clinical variables and other prognostic factors such as HA and bile acid levels to predict the risk of fibrosis and cirrhosis which might contribute to the progression of disease into HCC. Therapeutic interventions that ameliorate abnormal cholestasis biomarkers could be another element in the treatment of patients with CVH. Specifically, designed trials are needed to assess the impact of ameliorating abnormal cholestasis biomarkers on the prognosis of patients with CVH.

## Methods

### Study design and population

This was a prospective, cross-sectional, observational study based on medical records and primary data collection from patients with CVH (HBsAg negative) who were admitted to the infectious disease wards in five hospitals across China between February and September 2013. The hospitals were randomly selected from five provinces, municipalities or autonomous regions across China.

Adult patients with CVH undergoing treatment at the study hospitals who were deemed suitable for discharge based on clinical judgement by their treating physician were enrolled. Patients with established fibrosis/cirrhosis and coincidental hepatocellular carcinoma; coinfection with HCV and/or HIV, undetectable viral load by PCR; and factors which raise the liver enzymes such as alcoholism (>20 g/day for women, >40 g/day for men); acute hepatitis; non-alcoholic steatohepatitis; autoimmune disorders; congestive heart failure; glycogen storage diseases and use of immunosuppressive or hepatotoxic drugs were excluded. The study was approved by the ethics committee of Beijing Ditan Hospital, China-Japan Union Hospital, Guangzhou Eighth People’s Hospital, Huashan Hospital and Sichuan Provincial People’s Hospital. All patients provided written informed consent prior to their participation in the study. The study was conducted in accordance with the International Conference on Harmonization guidelines, and the Declaration of Helsinki. The reporting of this study conforms to the STROBE statement^25^.

### Study assessments

Patient demographics and medical history, including laboratory examinations like total bilirubin (TBIL), hyaluronic acid (HA) and type IV collagen levels measured during hospitalisation, were collected from the medical records. Levels of cholestatic enzymes such as alkaline phosphatase (ALP), GGT, aspartate transaminase (AST), alanine transaminase (ALT) and bile acids were measured before discharge. Also, TBIL, HA and collagen levels were measured before discharge to assess the presence of liver fibrosis. Patients were confirmed with liver fibrosis if the levels of these biomarkers exceeded the upper limit of normal reference range. Further, in order to estimate the severity of liver disease and risk of HCC, additional clinical and biochemical data required for calculation of Child-Pugh scores^26^, including the degree of ascites, grade of encephalopathy, albumin and TBIL levels, as well as prothrombin time. Child-Pugh scores were derived based on total score of all the indices. A total score of 5 to 6 was considered class A (well-compensated disease), 7 to 9 was class B (significant functional compromise), and 10 to 15 was class C (decompensated disease)^26^.

### Study endpoints

The primary endpoint was the proportion of patients with CVH who had elevation of cholestatic enzymes at the time of discharge. Secondary endpoint was the association between elevated cholestatic enzymes and liver fibrosis or disease severity.

### Statistical analyses

All analyses were performed using SAS version 9.3 (SAS Institute, Cary, NC, USA). Demographics, clinical characteristics, and baseline biochemical laboratory findings were summarized for the overall study population using descriptive statistics. Univariate analysis was performed to compare the demographic and clinical characteristics of patients with normal and elevated cholestatic enzymes. Also a multivariate analysis was performed by adjusting the relevant factors (such as age, gender, disease duration, total bilirubin, AST, ALT, bile acid, albumin, and prothrombin time) that could affect the cholestatic enzyme levels. Further, the association of risk of liver fibrosis and disease severity (measured in terms of Child-Pugh class) with elevated cholestatic enzyme status was estimated using multivariate logistic regression analyses after adjusting the relevant concomitant factors (such as age, gender, disease duration, HA and type IV collagen, AST, ALT, and bile acid). All the comparisons between groups used the Student *t* test or the Chi-square test, where appropriate. Missing data was excluded from analysis. A p-value of less than 0.05 was considered statistically significant.
